# Creating inclusive Performing Arts practices for development of youth with disabilities: A critical ethnographic study

**DOI:** 10.4102/ajod.v10i0.753

**Published:** 2021-06-30

**Authors:** Marlene le Roux, Harsha Kathard, Theresa Lorenzo

**Affiliations:** 1 Inclusive Practices Africa, Department of Health and Rehabilitation Sciences, University of Cape Town, Cape Town, South Africa; 2 Division of Disability Studies, Department of Health and Rehabilitation Sciences, University of Cape Town, Cape Town, South Africa; 3 Division of Communication Sciences and Disorders, Department of Health and Rehabilitation Sciences, University of Cape Town, Cape Town, South Africa; 4 Department of Health and Rehabilitation Sciences, Faculty of Health Sciences, University of Cape Town, Cape Town, South Africa

**Keywords:** Performing Arts, social inclusion, livelihoods, community-based rehabilitation, inclusive development, disability, youth development

## Abstract

**Background:**

Youth with disabilities are a marginalised group in society. This marginalisation traps them and prevents their full participation in social and economic development.

**Objective:**

This study sought to understand how exposure to the Performing Arts facilitates the inclusion of youth with disabilities.

**Methods:**

The study adopted a qualitative research approach, utilising critical ethnography. Primary data consisted of three focus group discussions with youth with disabilities, and an in-depth interview with a performer with disability. Thematic data analysis was conducted.

**Results:**

Four themes emerged. Theme 1, *Blown away*, shares the experiences of youth who attended Artscape Theatre. Theme 2, *I can do it, you can do it*, describes their career aspirations. Theme 3, *Embracing hope*, identifies the social and life skills learned through visited Artscape. Theme 4, *Long way to go*, presents the factors that influence the participation of youth with disabilities in the Performing Arts. While their experiences are diverse, and their impairments are unique, contact with the Performing Arts supported social and economic inclusion, and triggered empowerment of youth with disabilities. Insufficient accessible and available transportation is the most notable barrier to accessing development opportunities.

**Conclusion:**

Exposure to the Performing Arts provides important skills development and social opportunities for disabled youth. It is up to the ‘keepers’ of the Performing Arts – those in administration and management – to realign the Performing Arts in a way that can best benefit everyone.

## Introduction

The Artscape Theatre Centre in the Western Cape province of South Africa has an active audience development and education department housed in the Inclusive Arts Unit (IAU). The objective of the IAU is to use the Performing Arts as a vehicle to facilitate social transformation and build bridges across the divides, which separate communities, and to enable new potential audiences to access the theatre and the Performing Arts. A further aim was to nurture future generations of patrons through school and youth programmes, offering them opportunities to participate in productions and training. It is important to acknowledge that Artscape stands as a symbol of South Africa’s apartheid legacy as it was originally designated as a space for white people only. The IAU aims to undo the wrongs of the past by transforming Artscape into a place of hope and a home for artists and patrons of all backgrounds. This transformation includes artists and patrons with disabilities.

Historically, arts in South Africa have been used to give a voice to the voiceless. An example of this intention is the many underground ‘struggle plays’, which were written during the apartheid era. The lead author of this article also attests to the positive influence of involvement in the Performing Arts as a black woman with disability who grew up in a disadvantaged, rural community during apartheid. She describes her involvement in youth choirs as an experience of finding her voice. This experience boosted her confidence and helped to socialise her into society without the usual struggle and prejudice, which many persons with disabilities face. Opportunities to participate in the Performing Arts also assisted in enhancing her world views and reducing her fears and anxieties, thus making the outside world a more liveable place for her. Her involvement in Performing Arts enabled her to build relationships and helped her to make sense of both her disability and her unique abilities. This sense-making and relationship building may not be easy for youth with disabilities who continue to face complex challenges.^1^

Max-Neef (2009), a Chilean economist, has pointed out that development is about people, not objects; in other words, it is people centred. He identified nine fundamental human needs, namely, subsistence as priority, identity, affection, protection, creation, understanding, participation, freedom and idleness. These needs are non-hierarchical and inter-related, and are the same in all cultures and across historical periods. Any of these needs that are unmet may lead to deprivation, but they become a resource in themselves. For youth with disabilities, adequate care, medical equipment and rehabilitation, accessible buildings and transport, as well as emotional support are single or synergistic satisfiers of human needs for social and economic development (Lorenzo et al. 2018). In addition, youth with disabilities need access to adequate education and services in order to develop intellectually (Moyne 2012; Oliver & Sapey 2006). As one of the most marginalised groups in the society, their rights to economic opportunity and social development need to be vigorously protected through national policies and actively implemented in the local context. Confronting human poverties through the maximisation of local resources and social spaces can facilitate friendships and networks that provide opportunities for relationship building, so that a person who is different from you can be seen as equal.

This study focuses on the experiences of youth with disabilities of accessing the Performing Arts and explores the ways in which exposure to the Performing Arts creates opportunities for social and economic inclusion. Art influences the society by changing opinions, instilling values and translating experiences across space and time. Art can have an impact on an individual’s sense of self. The Disability Arts Movement empowers persons with disabilities to claim the right to be ‘equal but different’, as expressed in the affirmative model of disability (Swain & French 2000). This movement, which was initiated by activists, artists and creatives in the late 1970s, campaigned for the civil rights of people with disabilities and fought against their marginalisation in Arts and culture. Stöckl (2015:42) concluded that the Disability Arts Movement ‘emphasises the pride that disabled people feel: a pride that is sometimes lacking because of the social stigma that still prevails’. The Affirmative Model of Disability was suggested by Swain and French (2000), who advocated for a:

[*N*]on-tragic view of disability and impairment which encompasses positive social identities, both individual and collective … grounded in the benefits of lifestyle and life experience of being impaired and disabled. (p. 569)

They further argued that the Performing Arts open up a space for expression and, at the same time, the creation of images of pride and strength, in contrast to ideas of helplessness and dependency. The Performing Arts is an integral part of culture, any culture, and remains a stalwart of change.

## Methods

This study explored how the youth with disabilities who were exposed to theatre performances, through visiting the theatre or attending a theatre workshop, were influenced in terms of becoming aware of possibilities for social and economic inclusion. A qualitative research approach was adopted using a critical ethnographic approach as a research design. Critical ethnography investigates the culture, community and everyday circumstances of participants – what is and what could be (Thomas 1993). It involves seeking to uncover not only sociocultural knowledge about a group but also patterns of social injustice. The apartheid system in South Africa left many deep scars. There is a need to explore the role it played with regard to the social exclusion and marginalisation of specific communities, especially black youth with disabilities. Boylorn and Orbe (2014:15) asserted that critical ethnographers are interested in the ‘politics of positionality’, where researchers expose their own privileges, in addition to marginalisation, and ‘take responsibility for [their] subjective lenses through reflexivity’. As a researcher with a disability, the lead author of this study favoured critical ethnography as a means to address the past practices of discrimination and bias, and to develop new strategies to support the socio-economic inclusion of youth with disabilities.

In her earlier role as development coordinator in IAU, the lead author worked closely with disadvantaged persons. Additionally, in her book entitled *Look at Me* (Le Roux 2008), she collaborated with 25 other South African women with disabilities, documenting their personal experiences, perspectives and aspirations. Through the use of critical ethnographic approach, including the lead author’s proximity to youth with disabilities and to the theatre, as well as her positionality as a researcher with disability, she was able to critically analyse the experiences of youth with disabilities attending performances at the Artscape Theatre Centre.

### Sampling and participants

Participants in this study were recruited from poor, disadvantaged, black and coloured^2^ communities in the Western Cape province, South Africa. These communities continue to be impacted in the aftermath of apartheid as evident in inadequate resources, a lack of affordable and accessible transport, poor service delivery and spatial inequality (Le Roux 2018; Lorenzo 2008). The study involved three focus group discussions. Group 1 included six learners from a tertiary training college for the deaf who attended a production of an Afrikaans Set work^3^ (see Table 1). Group 2 included seven Grade 12 learners from a high school for the deaf who attended the same production (see Table 2). These two institutions deal with similar challenges and opportunities related to disability inclusion, and to providing skills and education to these young learners with disabilities. Group 3 included six audience members with a disability who attended an event at Artscape (see Table 3). In addition, an in-depth interview was conducted with a female dancer with disability to gain insights into her experiences of exposure to Performing Arts. A purposive sampling strategy was used to select participants.

**TABLE 1:** Participant group 1: Tertiary training college for the deaf.

Name	Age	Gender	Disability
Priya	18 years	Female	Deaf
Stephanie	21 years	Female	Deaf
Claudia	20 years	Female	Deaf
Viwe	21 years	Female	Deaf
Brandon	21 years	Male	Deaf
Hayley	20 years	Female	Deaf

*Source:* Le Roux, M., 2018, ‘There is a place in the sun for people with disabilities within the Performing Arts: Exploring how interaction with the performing arts may facilitate the social and economic inclusion of youth with disabilities’, Unpublished MPhil in disability studies thesis, Faculty of Health Sciences, University of Cape Town, Cape town

**TABLE 2:** Participant group 2: Secondary high school for the deaf.

Name	Age	Gender	Disability
Koos	20 years	Male	Deaf
Erik	18 years	Male	Deaf
Howard	18 years	Male	Deaf
Nathan	18 years	Male	Deaf
Adele	18 years	Female	Deaf
Rene	18 years	Female	Deaf
Liezel	18 years	Female	Deaf

*Source:* Le Roux, M., 2018, ‘There is a place in the sun for people with disabilities within the Performing Arts: Exploring how interaction with the performing arts may facilitate the social and economic inclusion of youth with disabilities’, Unpublished MPhil in disability studies thesis, Faculty of Health Sciences, University of Cape Town, Cape town

**TABLE 3:** Participant group 3: Artscape youth group.

Name	Age	Gender	Disability
Nonzuzu	29 years	Female	Bipolar
Yinka	30 years	Female	Osteogenesis Imperfecto
Chidera	35 years	Female	Osteogenesis Imperfecto
Rufaro	35 years	Female	Paraplegic
Dylan	24 years	Male	Osteogenesis Imperfecto
Tariro	24 years	Female	Blind
Vuyo	32 years	Male	Ciphernia† (Left)

*Source:* Le Roux, M., 2018, ‘There is a place in the sun for people with disabilities within the Performing Arts: Exploring how interaction with the performing arts may facilitate the social and economic inclusion of youth with disabilities’, Unpublished MPhil in disability studies thesis, Faculty of Health Sciences, University of Cape Town, Cape town

† , His own explanation for his mobility impairment from polio.

Participants were youth, that is, their ages ranged from 18 to 35 years, as defined by South Africa’s *Youth Commission Act* of 1996. They self-identified as male or female; had self-described sensory, mobility, mental or psychosocial disabilities; and were willing to share their experiences. Participants were from bilingual backgrounds with English as one of their languages. Participant information is summarised in Tables 1–3. The names provided in the table are pseudonyms that were used to protect the privacy of the participants.

### Data gathering methods and data analysis

The primary data gathering method involved three focus group discussions youth with disabilities, an in-depth interview with a performer with disability and reflective journaling by the researcher. The use of focus group discussions as a data gathering tool was an effective way for investigating the limiting factors affecting the youth with disabilities. A focus group is a relatively small gathering of individuals who assemble in one location to discuss topics specified by a researcher (Smithson 2000). The focus group format effectively prompts discussion between participants, potentially generating a diverse blend of perspectives and suggestions (Marshall & Rossman 2006). The focus groups centred on the participants’ experiences of attending a performance, event or workshop at Artscape. In addition, the focus groups explored participants’ views regarding the influence of the Performing Arts on their social and economic inclusion. Although the researcher intended to recruit participants from diverse race, gender and impairment backgrounds for the focus groups, this was not logistically feasible. Therefore, each focus group comprised participants who were from the same institution. The in-depth qualitative interview with the performer with disability explored her experiences of inclusion in the Performing Arts as a marginalised performer. As a critical ethnographer, the lead researcher used reflective journaling as a means to be self-reflexive through the research process. She documented her positionality, thoughts, emotions, critical incidents and learnings to strengthen the data collection and analysis process.

Data analysis began with the transcription of all digitally recorded data. Creswell (2007) suggested that in reading through data and gaining familiarity, one can start the process of understanding it. Thematic analysis, as described by Bowen (2009) and Braun and Clarke (2006), was used to identify recurring themes and patterns in the focus group interactions, specifically focusing on a critical ethnographic concern with patterns of social injustice. Braun and Clarke (2006:78) viewed thematic analysis as a flexible tool for research, ‘which can potentially provide a rich and detailed, yet complex, account of data’. Thematic analysis enables researchers to make sense of data in accordance with their specific interests and within a broader methodological framework (Braun & Clarke 2006). The researcher established a manual coding system to effectively categorise responses (Bowen 2009; Braun & Clarke 2006). Any additional comments were included as a means to contextualise focus group transcripts. Review and constant comparison enabled the researcher to place codes into categories and subcategories, and the verification of themes and categories was carried out up to the point where saturation was reached. Pseudonyms were used to identify participants and their responses, ensuring that confidentiality and privacy were not compromised.

This research study adhered to Lincoln and Guba’s (1985) strategies of credibility as the author carried out member-checking with the participants. Transferability was achieved through providing thick description of the context of the study participants. The researcher improved the dependability of the study by maintaining an audit trial of the research process and analysis. The researcher engaged in critical dialogue with her peers with disabilities and her supervisors to assist with confirming her analysis. The researcher ensured that the participants were informed of all the potential benefits and hazards of the study.

## Findings

The following four themes related to disability inclusive development emerged from the data:

- Theme 1: ‘Blown away’ reflects the amazement experienced by youth when they see a performance for the first time.
- Theme 2: ‘I can do it; you can do it’ describes the career aspirations of youth with disabilities.
- Theme 3: ‘Embracing hope’ explores the social and life skills learned through exposure to the Performing Arts.
- Theme 4: ‘Long way to go’ outlines the factors that influence the participation of youth with disabilities in the Performing Arts.

### Theme 1: Blown away

The first theme is Blown Away (Samantha, Interview, March 2017), which describes the experiences of youth with disabilities who attended a performance or an event at Artscape. For the majority of the participants, regardless of whether it was their first visit to the Artscape Theatre Centre, the mere fact of attending an event was an overwhelming experience that evoked numerous feelings:

‘When I entered Artscape it was unbelievable for me to see how they were acting, how the characters were portrayed. I could fit into what was happening because there was an interpreter and so I could understand everything. Also, with the changing of the lights I could feel it and be part of it. It was like being in a 4D movie.’ (Howard, Group 2, March 2017)

The participants from groups 1 and 2 were truly astonished after attending a performance of a set work at the Artscape Theatre Centre. They were able to understand what was unfolding and what the actors were actually saying as a sign language interpreter was present on the stage during the performance: ‘the best part was that there was an interpreter for all [deaf persons attending], that could contribute to helping all who attended understand the set work’ (Nathan, Group 2, March 2017).

Non-formal education through attending an event or performance allows for youth with disabilities to engage with others and learn new skills and abilities through participative interaction: ‘It’s a very fun and exciting space to be in because you interact with different people and you always leave with new friends’ (Chidera, Group 3, March 2017). Additionally, the performance was educational:

‘You can always learn something new from every production. Each production has a theme and that theme always provokes different emotions and ideas. It can either be something personal or something that’s out there, but you always leave with something that speaks to something else.’ (Yinka, Group 3, March 2017)

The performance of a set work also acted as an assistive educational aid for educational institutions as learners are exposed to performances that portray texts that they are studying as part of their curriculum:

‘It is an extremely wonderful experience to take learners to Artscape, especially when it’s to my advantage because it addressed work we need to do in class. So, it really is very valuable, to see something in writing and then be able to see it being performed.’ (Educator, Group 2, March 2017)

Performing Arts offer a space for lifelong learning as one leaves each performance and event having learnt something. Arts education is vital to ensure holistic development as it enhances an understanding of the world, exposing individuals to different views, the many personalities that make up the world, the many faces, forms, shapes and colours. This can show youth with disabilities that they are not alone and help them to make sense of their own abilities.

### Theme 2: I can do it, you can do it

This quote (Brandon, Group 1, 2017:21) reflects the second theme, which addresses how being at Artscape may influence the career aspirations of youth with disabilities. There were two sub-themes, namely, ‘[c]reative possibilities and opportunities’ and ‘[i]nspired’.

Through exposure to new things outside of what they are normally used to, youth with disabilities are able to build not only their confidence but also resilience as they are encouraged to explore and discover their own unique personal abilities. One of the participants spoke very openly about how, if opportunities were not available, one should go out and create them. She mentioned how after an existing organisation she had joined closed down, she realised the need to form a new organisation for youth with disabilities, a place where they could come together and share experiences, network around possible employment opportunities and engage socially. Prompted by this realisation, she went out and registered her own organisation and had the following to share with the group:

‘They need to face the challenges that’s out there. Accepting the fact that you are disabled, you can’t go there, and you can’t do this because of all these stumbling blocks. It’s not gonna help, it’s just keeping you back. So, if we force our way in masses then that’s how we gonna change people’s mindsets, in living our lives the way we want to.’ (Chidera, Group 3, 2017:10)

*Inspiring* emerged as a category that revealed how youth took courage in pursuing their career dreams. Performing Arts creates opportunities for engagement and interaction that were inspirational and empowering on many levels. These contribute to the process of developing self-determination. Many of the participants in Group 3 already had employment. Their main concern was how to retain employment in a career that they were passionate about, and which was economically viable. All of these young people came from disadvantaged areas in Cape Town and had careers within Performing Arts. Some participants were unemployed, and they participated in informal Arts-related activities in their communities: ‘I’m a singer by nature but the acting and everything I did with my friends in Khayelitsha^4^’ (Tariro, Group 3, March 2017).

### Theme 3: Embracing hope

This emerged as the third theme, which addressed the manner in which social and life skills are learnt by attending a performance or visiting the Artscape Theatre Centre. Two sub-themes, namely, *Connecting socially* and *Gaining self-mastery*, were identified.

For all participants, social cohesion in a disability context meant being able to be like any young adult or teenager – to go out and enjoy life with their peers. Coming to Artscape was a whole new experience:

‘They think that is life and then when they come to the theatre it’s a whole new experience for them, it’s exciting, it’s a different experience, it’s something that they [have] never seen or experienced before. So, by creating opportunities like these for young people, I think it does start changing the way we live and our societies. It starts giving young people hope and it makes them realise that they have the skills, they have the tools but it’s just a matter of working on them and realising that “I am worthy” and “I can do anything I want” and then “where to from here”.’ (Samantha, Interview, March 2017)

Samantha expressed how attending an event or performance instilled a feeling in youth with disabilities that they could be part of the bigger world. They could see themselves enjoying an experience at the same event and venue as any other person.

This finding suggests the importance of access to recreation as part of inclusive development. An inclusive development practice approach advocates for the Artscape Theatre Centre, and other spaces of engagement, to develop systems that recognise a caregiver or personal assistant as integral to accessibility for the person with disability. For example, when purchasing tickets to an event, the caregiver needs to be allowed to enter free of charge.

Being amongst peers and with people without disabilities created a sense of belonging in youth with disabilities, and developed their confidence to branch out, disrupting the stereotyping and exclusion of people with disabilities. Youth with disabilities develop confidence and self-esteem to overcome the fear and anxieties associated with life in a non-disabled world through interactive spaces. Chidera commented: ‘For me, it’s a very fun and exciting space to be in because you interact with different people and you always leave with new friends’ (Chidera, Group 3. 2017:12).

In order for a young person to be included and to contribute to the economy of life, they need support to take ownership of their circumstances, which enables them to make life choices (Galvaan 2010). Social participation, positive identity, life skills and creativity offered through exposure to the Performing Arts contribute to greater progress in the social and economic inclusion of youth, especially youth with disabilities.

Most of the participants clearly described how opportunities to interact socially have positively influenced their identity:

‘When I got involved with Performing Arts, meeting new people on a daily basis, working with different people all the time, I’ve become more confident and I’ve learnt that there’s more to life than just one way of doing things.’ (Nonzuzu, Group 3, 2017:15)

Through connecting socially within the interactive spaces provided by attending an event, youth with disabilities are given the opportunity to experience something new, whilst also learning and developing new skill sets that are vital for enhancing their chances for different forms of employment. Opening up an exciting space for them to form new friendships and lasting networks could also potentially lead to avenues in exploring employment opportunities.

### Theme 4: A long way to go

Exposure to the Performing Arts has clear benefits for youth with disabilities. In contrast, this fourth theme identifies the social and environmental factors that influence the participation of youth with a disability in the Performing Arts. One of the sub-themes, *Freedom to travel,* was identified as essential to people-centred development. Without accessible and flexible transport systems, youth with disabilities are not fully able to participate and engage in the society. Difficulties in accessing public and private transport systems exclude youth with disabilities from the freedom of attending a performance and the opportunity to engage with others outside of restricted timeframes.

Public transport was a frequent issue raised in the focus group discussions and the in-depth interview. In order for youth with disabilities to be able to do anything – interact with society and environments, and have access to work or entertainment – transport plays a crucial role. Samantha shared a story of an acquaintance as an example of just how challenging it was to make use of public transport for a person with a disability:

‘There was a boy in a wheelchair who lives in Khayelitsha and he used a taxi one day to come to Artscape, and he arrived two hours late. We asked him what the experience was like for him and he said that he had to wait for two hours to get into the taxi because, first of all, there was no one who was willing to help him. He had to wait until someone came along who was actually willing to assist him out of his wheelchair and into the taxi, and then having to pay another seat in the taxi. So ja, I think we still have a long way to go with public transport.’ (Samantha, Interview, 2017:3)

Youth with disabilities regularly require assistance from friends, family members and caregivers to travel on public transport, and often have to endure added challenges in the form of extra costs to be able to make use of different modes of transport.

The participants in Group 3 also expressed how difficult it was to access public transport routes within their communities. This is an issue directly linked to the apartheid legacy. They often require assistance as these public transport routes are frequently difficult, even impossible, to use, particularly because of the fact that their assistive devices (such as wheelchairs) do not allow them to easily manoeuvre within the built environment: ‘[t]he challenge is I can’t go alone to take the transport because of the roads and it’s also not wheelchair-friendly for me to be able to travel alone’ (Chidera, Group 3, 2017:19).

Some of the participants from the same focus group mentioned that the Dial-a-Ride taxi service, which specialises in offering transport to people with disabilities, is an extremely unreliable mode of transport:

‘My mode of transport is dial-a-ride, which means that I have to always have bookings in advance. So, if I want to go somewhere it has to be seven days in advance for me to make the booking. So, if there comes up an event tomorrow, I can’t attend because I haven’t made prior bookings to go to the event.’ (Dylan, Group 3, 2017:19)

Participants also pointed out that the operating hours of public transport systems in their areas were often not convenient, making it difficult for them to access opportunities, such as social functions and performances at the Artscape Theatre, which are often held in the evening.

Private transport revealed a different dimension to the transport issue. For the participants from groups 1 and 2, access to transport for attending social events at the Artscape Theatre Centre and elsewhere was not problematic. These institutions have full-fledged transport systems to accommodate their learners within their secured learning environment structures. Youth who attend special schools enjoy, for the limited years of their schooling, the conveniences of established infrastructure and accessible transport, as well as organised opportunities for development. We must consider what happens once they left the school, as the infrastructure and opportunities are no longer available to them. Unfortunately, this translates into people with disabilities taking steps back instead of forward as these individuals no longer have facilitated access to theatres and other social spaces.

Access to privately owned modes of transport posed a problem for some of the participants, especially the black and coloured learners, whilst the majority of the white learners easily accessed privately owned modes of transport: ‘[c]urrently we are busy building up my own car, so that I will be able to drive here on my own next semester’ (Koos, Group 2, 2017).

In this study, the continuous impact of the apartheid legacy is clearly visible, specifically linked to the issue of freedom of movement and access to spaces.

## Discussion

In this study, a critical ethnographic approach enabled the exploration of the specific experiences of youth with disabilities accessing the Artscape Theatre Centre. It explored the efforts on the part of this post-apartheid institution to become a responsive and inclusive Performing Arts Centre for patrons with disabilities. It also allowed for reflection on past injustices, and the manner in which persons with disabilities continue to be impacted by the apartheid legacy. Whilst the experiences of youth with disabilities are diverse, and their impairments are unique, contact with the Performing Arts supported social and economic inclusion and triggered their empowerment. Unfortunately, youth with disabilities, especially those further disadvantaged by the legacy of apartheid, experience barriers to accessing these opportunities, most notably insufficient transportation.

Education is a basic human right. An inclusive development practice approach to disability attempts to do away with attitudinal, physical and communication barriers by suggesting that the acquisition of knowledge is based on not only the numeric and literacy competence but also visual and artistic competences. Similarly, in advocating for an inclusive approach that embraces disability, rehabilitation services should not focus exclusively on issues of health, but it needs to embrace social and Arts interactions as well as political and economic inclusion. Importantly, inclusion is more than integration, as it involves bringing previously neglected and under-represented persons into a participatory role and the decision-making process, effectively ensuring their ownership in the process and its results. This approach to inclusion enables people to find ways to build bridges together so that barriers to participation related to attitudes, the natural environment, services and systems, support structures, and products and technology (WHO 2001) are removed. These environmental factors are synergistic satisfiers of human needs (Max-Neef 2009). Where they are not provided, the participation of youth with disabilities in the Performing Arts is limited, thus leading to deprivation and human poverties. The findings also reveal that emotional support is vital. It allows for a care-based ethic to develop where sharing can occur based on the notion of interdependence and connectedness (Kittay 2011). It is not only the environment that needs to be conducive to participation by youth with disabilities but also the need for care has to be acknowledged. It is care that has potential to build the resilience of youth with disabilities.

The lead author has developed a concept of inclusion as a ‘*laslappies kombers’*, which is an Afrikaans term. It describes the multi-coloured blankets made from cut material (patchwork quilt) by the women in her rural town, Wellington, in the Western Cape province of South Africa. It is a term the lead author developed to expand the understanding of intersectionality in the disability inclusion workshops that she facilitates. It is a construct of interwoven identities, which makes up the fabric of society (Le Roux 2018). The blanket’s fabric is strong, beautiful and useful when it includes everyone. Inclusion is a process of weaving together the fabric that makes up society, recognising the value of each thread.

Such an inclusive approach may provide a platform where youth both with and without disabilities get to know one another, where diversity is embraced and social anxieties are reduced. Inclusion must be person centred, thus enabling persons with disabilities to resist social stereotypes, confront their fears, build a positive identity with creativity and curiosity, acquire skills and confidence, and experience social participation. Such processes pave the way to greater social, political and economic inclusion. This approach is important for advancing South Africa’s democracy. As the lead author states:

‘We were all brutalised by apartheid and colonisation – so we must all find a new way together. Each of us has a story to tell, and in our story whether of being victim or perpetrator, we can both make sense of ourselves and with each other. We need to draw on our collective wisdom in Africa to honour our humanity. We all need to find a way through which we can build inclusivity in our homes, communities, schools, universities and workplaces. We cannot isolate disability as a separate issue and think we are inclusive. We need a nuanced approach. There are so many ancestries across the world. We speak from our place and being in Africa.’ (pers. comm., Le Roux 2018)

In her thesis, she has also expanded on an African inclusive model of disability, which reflects the unique diversity of the languages, race, gender and cultures that persons with disabilities come from (Le Roux 2018). In South Africa, there is a backlog when it comes to storytelling, whose stories are being told and by whom. We need to see the Performing Arts in the bigger picture of life and consider what exposure to the Performing Arts can do for a person, whether as a consumer or as a storyteller. We need to change the archaic narrative that Performing Arts is only for an elite group of people. In her role within the Performing Arts in South Africa, the lead author has formulated strategies for providing universal access to the Performing Arts. In exploring the barriers to participation faced by youth with disabilities, she questions the pillars of authority that support the status quo and expresses the hope of moving beyond the current practices to inclusive practices (Galvaan 2010; Noblit, Flores & Murillo 2004).

## Conclusion

This study concludes that youth with disabilities experience benefits from being included in the Performing Arts. It also draws attention to how systemic exclusion occurs through the combined effects of social, economic, educational, institutional and political systems. Inclusion is challenged in multiple ways. Youth, particularly from disadvantaged communities, are trapped in their schools through poor curricula, as well as have limited access to resources and friendships to help them socially. They remain on the margins and are isolated through persistent apartheid-era design and practices. At a personal level, they become disempowered and urgently need a stepping stone through which to become engaged in society.

However, the study reveals how changes can be effected by key actors in the Performing Arts, communities and youth with disabilities themselves to create opportunities, which facilitate social and economic inclusion. Exposure to the Performing Arts can provide essential skills development and social opportunities for youth with disabilities. It is up to the ‘keepers’ of the Performing Arts – those in Arts administration and management – to realign the Performing Arts in a way that can best benefit everyone.

Inclusion as an aspirational value can be imagined as a ‘*laslappie kombers*’, which represents the interwoven identities of individuals who make up the fabric of society. Society will be strong, beautiful and useful when it recognises and values everyone that creates a cohesive society. In offering this view of inclusion, which values community connectedness, we provide a means to inspire authentic ways of engaging with disability inclusion. This view may have resonance in contexts where people experience oppression and marginalisation.

### Study implications

Finally, based on the research presented, this article makes the following recommendations:

- Promote flexible, affordable and accessible transport to enable youth with disabilities to participate in events, such as those in the Performing Arts.
- Encourage special needs schools to liaise with mainstream schools to work on projects together, especially in the Performing Arts or by attending each other’s social and educational events.
- Integrate Performing Arts studies into an inclusive teaching and learning training curriculum at special schools.
- Adopt inclusive practices at Performing Arts institutions to practically facilitate the safe inclusion of youth with disabilities. This inclusion needs to ensure safe access, providing staff training on inclusion of persons with different types of impairments and fostering a culture of inclusion within the institution.

## Footnotes

1 For the purposes of this study, the UN Convention on the Rights of Persons with Disabilities’ definition of disability will be used. Persons with disabilities include those who have … equal basis with others (UN, 2006).

2 ‘Coloured’ is a term used in South Africa to refer to individuals of mixed parentage. Whilst it was a racial category imposed under the apartheid regime, it remains used in positive terms – denoting a particular identity and referring to aspects of culture.

3 Referring to a piece of literature studied as part of the language curriculum.

4 Khayelitsha is referred to as a ‘township’ on the outskirts of the City of Cape Town. Originally designated as an area for black persons during apartheid, it remains an area of extreme poverty, facing a lack of service delivery and infrastructure, large-scale unemployment and crime.
